# Bonds over Electrons: Proton Coupled Electron Transfer at Solid–Solution Interfaces

**DOI:** 10.1021/jacs.2c10212

**Published:** 2023-03-21

**Authors:** James M. Mayer

**Affiliations:** Department of Chemistry, Yale University, 225 Prospect Street, New Haven, Connecticut 06520, United States

## Abstract

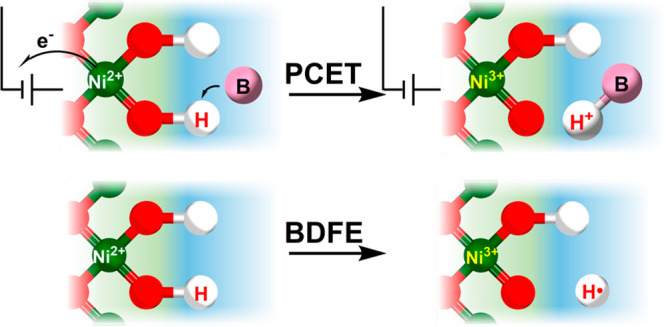

This Perspective argues that most redox reactions of materials at an interface with a protic solution involve net proton-coupled electron transfer (PCET) (or other cation-coupled ET). This view contrasts with the traditional electron-transfer-focused view of redox reactions at semiconductors, but redox processes at metal surfaces are often described as PCET. Taking a thermodynamic perspective, transfer of an electron is typically accompanied by a stoichiometric proton, much as the chemistry of lithium-ion batteries involves coupled transfers of e^–^ and Li^+^. The PCET viewpoint implicates the surface–H bond dissociation free energy (BDFE) as the preeminent energetic parameter and its conceptual equivalents, the electrochemical *n*e^–^/*n*H^+^ potential versus the reversible hydrogen electrode (RHE) and the free energy of hydrogenation, Δ*G*°_H_. These parameters capture the thermochemistry of PCET at interfaces better than electronic parameters such as Fermi energies, electron chemical potentials, flat-band potentials, or band-edge energies. A unified picture of PCET at metal and semiconductor surfaces is presented. Exceptions, limitations, implications, and future directions motivated by this approach are described.

## I Introduction

Redox reactions at solid–solution interfaces are important in electrochemistry, (photo)electrocatalysis, corrosion, metal production and electroplating, pseudocapacitors, batteries, and other applications. These various areas have developed somewhat different conceptual models. This Perspective develops a common description for many of these processes as proton-coupled electron transfer (PCET), and its relative cation-coupled electron transfer. Examples are given for redox reactions at metal surfaces, at semiconductor surfaces, and of molecules, in contact with aqueous media or other solvents with significant proton activity. The broad PCET perspective prompts a reconsideration of some common descriptions of interfacial redox reactions, especially at semiconductor/solution interfaces.

This Perspective was in part inspired by a 2010 JACS Perspective by Allen J. Bard,^1^ “Inner-Sphere Heterogeneous Electrode Reactions. Electrocatalysis and Photocatalysis: The Challenge.” Over the last dozen years, such chemical/electrical energy conversions have taken on new urgency because of climate change. Some of the critical (photo)electrochemical reactions that new energy systems will need to catalyze are shown in eqs 1–4. 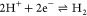
1
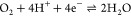
2
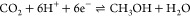
3
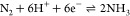
4These need to be catalyzed in both directions, at high rates, with high efficiencies, and using earth-abundant materials. Bard emphasized that such catalysis requires inner-sphere reactivity at the electrode surface, making and breaking bonds to surface atoms. This requirement follows from the typically extreme potentials needed to oxidize or reduce the neutral substrates by outer-sphere electron transfer (ET). Many inner-sphere reactions involve surface–hydrogen bonds. This motivates a PCET description, especially since eqs 1–4 are each PCET processes.

Many laboratories around the world are working on these challenges and probing redox bond making/breaking at solid/solution interfaces (e.g., refs (2−13)). This Perspective is far from comprehensive but rather aims to highlight the generality of PCET at interfaces and to develop the general principles and issues. The Perspective does not claim that all redox reactions at solid–protic solution interfaces are PCET, but the title “Bonds over Electrons” is meant to encourage a PCET way of thinking.

## II Traditional Models for Interfacial Redox Reactions

**Scheme 1:**
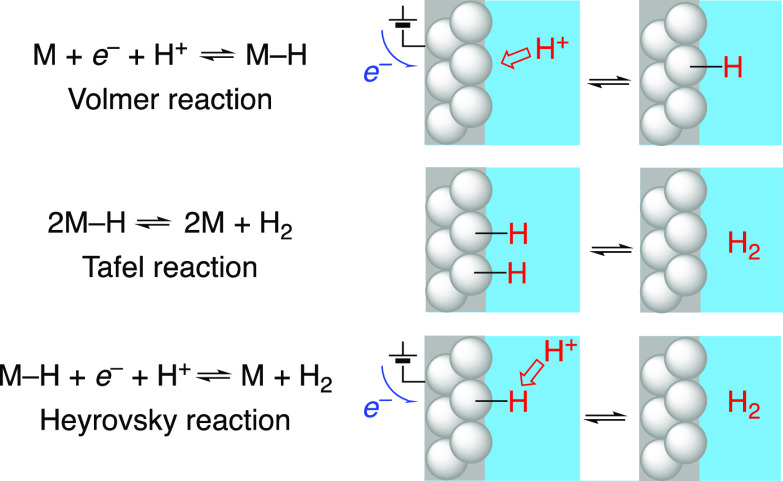
Elementary Steps in the Electrochemical Hydrogen Evolution and Oxidation Reactions (HER/HOR)

Interfacial, inner-sphere PCET reactions are not novel. PCET steps have been implicated in the mechanism of the hydrogen evolution reaction (HER) since the early 20th century.^14^ The Volmer and Heyrovsky steps in Scheme 1 explicitly involve e^–^ and H^+^, and the Tafel step involves the movement of hydrogen atoms that are implicitly e^–^ plus H^+^. These are the paradigmatic redox steps for catalytic and electrocatalytic processes at metal/solution interfaces, and they are all PCET.

**Figure 1:**
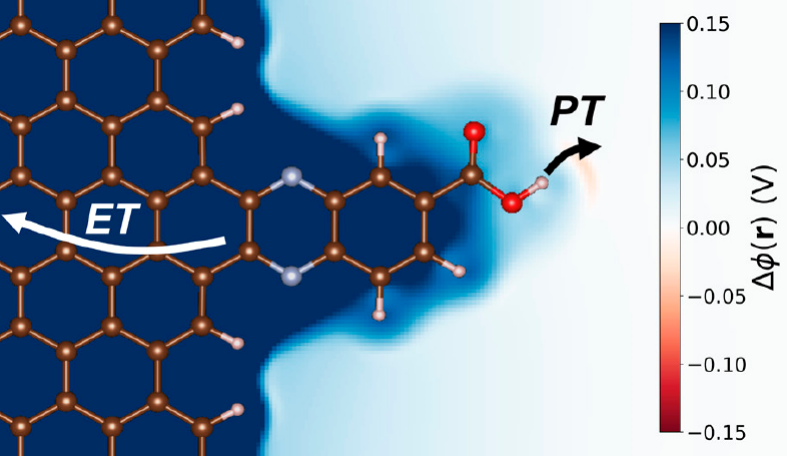
Schematic of the carboxylic acid-conjugated graphite surface, showing coupled ET and PT. The background colors represent the electrostatic potentials referenced to the point of zero free charge (PZFC). Reproduced from ref (15). Copyright 2020 American Chemical Society.

These concepts have recently been extended to carbon surfaces with attached acid/base sites by Surendranath, Hammes-Schiffer, and co-workers.^6,15^ When a carboxylic acid is strongly conjugated into the graphitic band structure, deprotonation is coupled to ET in a PCET process (Figure 1), and there is a clear electrochemical response. This is in essence the reverse of the Volmer reaction (Scheme 1). Yet when a single CH_2_ group is inserted between the carboxylic acid and the graphitic structure, breaking the conjugation and moving the carboxylate further into the solution, proton transfer (PT) becomes uncoupled from ET.^6,15^

In contrast with the widespread recognition of PCET reactions at metallic surfaces, redox reactions at semiconductor/solution interfaces have been predominantly discussed with a band-structure model.^16−21^

**Figure 2:**
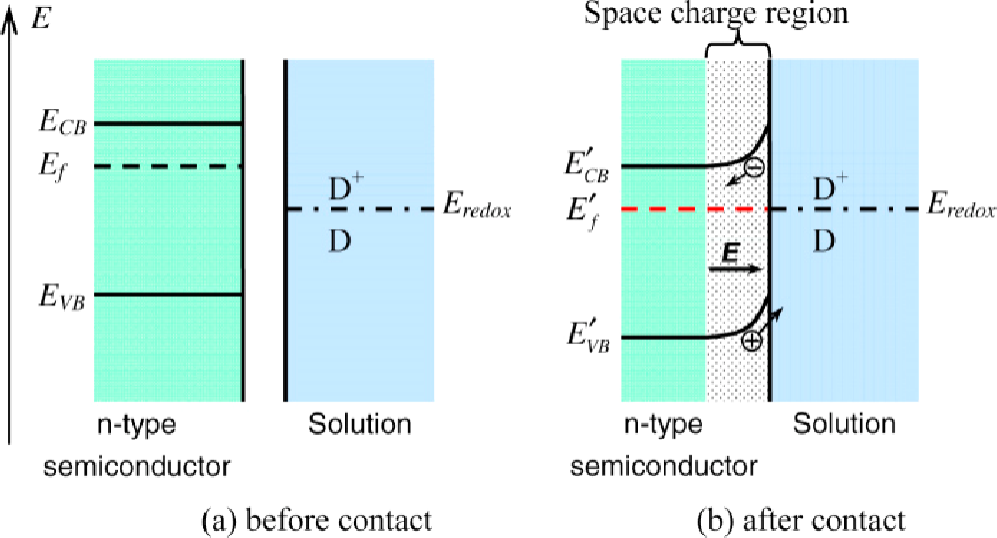
Contact of an *n*-type semiconductor with a solution containing a molecular redox couple D^+^/D. (a) Before contact and (b) after contact. Reproduced with permission from ref (23). Copyright 2013 Institute of Physics Publishing.

In a simple version of this electron-focused model, the separated semiconductor and solution have characteristic electrochemical and chemical potentials for the electron,  and *μ*_e_, respectively. These are defined by the Fermi energy in the solid and the reduction potential of an electroactive species in solution (Figure 2a).^22^ Bringing these phases into contact makes two potentials equal via movement of electrons and/or holes (*e*^–^/*h*^+^) into or out of the surface layer of the semiconductor. The solution potential remains essentially fixed because of the large excess of the solution molecular species over the surface *e*^–^/*h*^+^. The *e*^–^/*h*^+^ movement creates a space-charge region near the semiconductor surface, with an internal electric field (Figure 2b). This model follows directly from well-established solid-state physics of semiconductors, resembling the interface of *n*-type and *p*-type semiconductors in a diode. We will refer to this as the “Gerischer model.”

In this model, reduction of a semiconductor involves adding an electron to the conduction band (CB) (or a trap state close in energy). Similarly, oxidations add holes or remove electrons to/from the filled valence band (VB). Therefore, the thermodynamic energies of the CB and VB are key parameters of a material, for instance for driving solar fuel reactions such as eqs 1–4.

**Figure 3:**
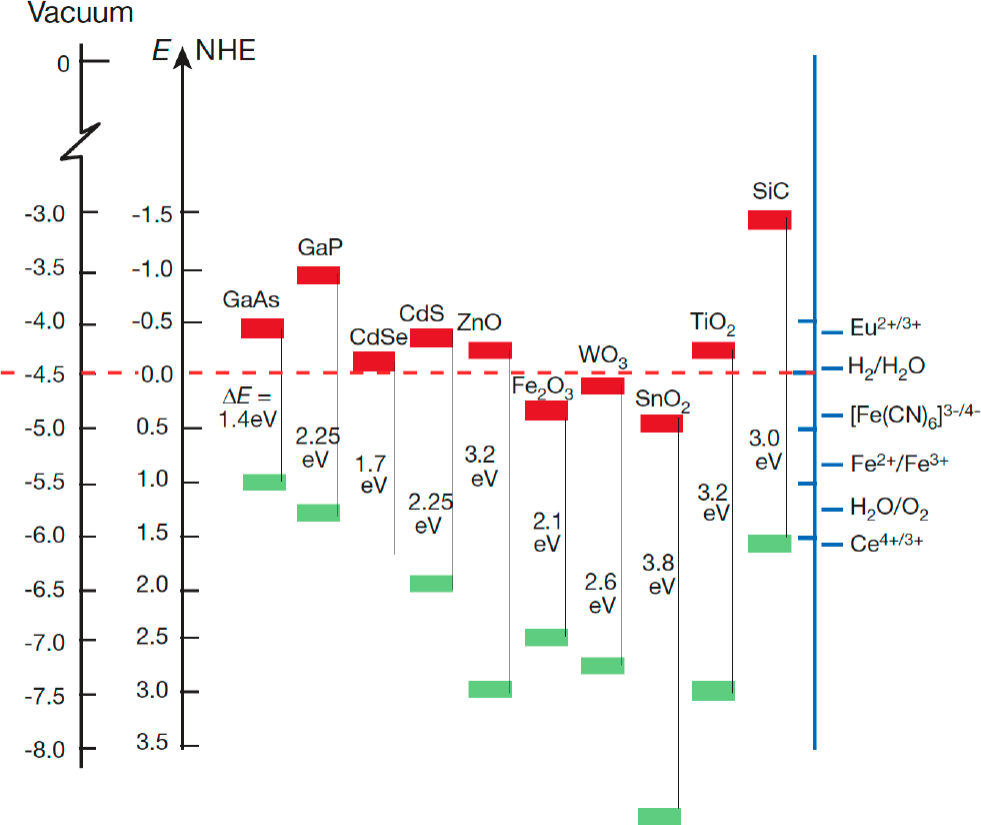
“Band positions of several semiconductors in contact with aqueous electrolyte at pH 1. The lower edge of the conduction band (red colour) and upper edge of the valence band (green colour) are presented along with the band gap in electron volts. The energy scale is indicated in electron volts using either the normal hydrogen electrode (NHE) or the vacuum level as a reference...” Figure and caption reproduced with permission from ref (24). Copyright 2001 Springer Nature.

The CB and VB energies for different materials in contact with aqueous solutions are often summarized in diagrams such as Figure 3(24) (examples go back to at least 1978^19,20^). Figure 3 shows band energies specifically at pH 1 versus NHE, but these are often shown at other pHs (e.g., pH 7 in ref (20)) or versus the reversible hydrogen electrode (RHE). These band energy diagrams are valuable but often misleading. For instance, the electrochemical potential scale should not be placed next to the work function in vacuum because the material surface at pH 1 is quite different than the same material *in vacuo*. Most semiconductor surfaces are significantly protonated at pH 1. The band potentials versus NHE move substantially with pH (see below) while the work function does not, as there is no pH *in vacuo*.

This electronic band-structure or Gerischer model has been central to semiconductor electrochemistry and photoelectrochemistry for half a century. It has been very successful and appears in multiple papers published daily.

Despite its success, this Perspective argues that the Gerischer model is incomplete in the ways that it is commonly used. The model implicitly assumes that the surface does not undergo any chemical change. Only the electrons move in response to the contact between surface and solution. This assumption is usually fine for the interface between two solids, but electrocatalysis is often done in corrosive media containing acids, bases, and electrolyte ions. As emphasized in Bard’s perspective, bonds are made and broken at the solid surface and redox changes occur in the material.^1^ Please note that we find no fault in the work of late Professor Dr. Gerischer, who was a giant in this field. He was completely aware that this model would not apply in a simple way to cases where ions bound to or intercalated into a solid (cf., ref (25)). We argue here that the Gerischer model is being applied to systems that do not follow its underlying assumptions. In our view, the typical −59 mV/pH dependence of semiconductor band edge energies with pH (see below) implicates surface chemical change, specifically proton binding and PCET. This changes the interpretation of Figure 3, as discussed below.

## III Introduction to PCET

Proton-coupled electron transfer (PCET) has become a significant theme in many areas of chemistry.^26−28^ Overlapping PCET research in inorganic chemistry, organic chemistry, biological chemistry, physical chemistry, electrochemistry, and chemical theory has enriched all of these areas. PCET generally refers to chemical processes whose rates and/or thermochemistry are affected by the movement of electron(s) and proton(s). The energy reactions 1–4 are examples of multielectron PCET, as are most biochemical energy conversions. In fact, the mitochondrial “electron transport chain” should really be called the proton-coupled electron transport chain.

**Scheme 2:**
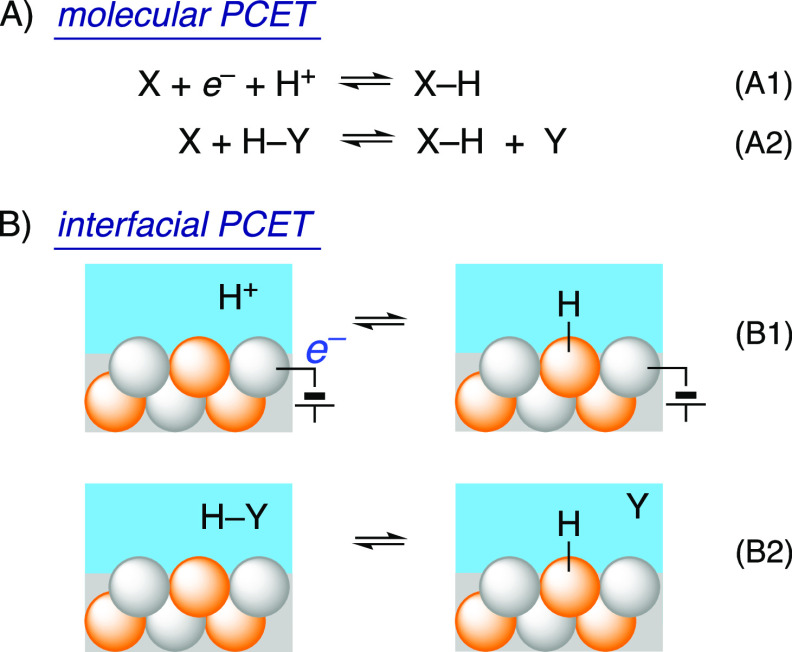
Schematic PCET Half and Whole Reactions with Molecules (A) and Materials (B), Illustrated for a Binary Material

These *n*H^+^/*ne*^–^ PCET processes proceed in many mechanistic steps. The protons and electrons can transfer in sequential steps or in the same kinetic step (the latter termed concerted proton–electron transfer, CPET).^25^ Within the class of CPET reactions, the *e*^–^ and H^+^ can transfer together as a hydrogen atom or they can transfer in one step but be physically separated in the reactants or products.^29^ For instance, a CPET reaction could involve an *e*^–^ from an external circuit via an electrode and a H^+^ from a solution reagent.^30,31^ Some of these cases are illustrated for molecules and interfaces in Scheme 2. The Volmer and Heyrovsky reactions in Scheme 1 can be considered examples of reaction class B1, while the Tafel reaction is closer to B2 (with Y = H also being bound to the surface). Even in a stepwise mechanism such as ET followed by PT, the two steps are thermodynamically coupled, as described in section V (and in our recent review of PCET thermochemistry^26^).

## IV Molecular and Interfacial PCET Stoichiometry

The study of chemical reactions begins with stoichiometry: the composition of the reactants and products and a balanced chemical equation. Stoichiometry is critical for electronic structure calculations, thermochemical analyses, yields, selectivity, and more. For this PCET Perspective, three kinds of stoichiometry are important: (i) the ratio of *m* protons to *n* electrons being transferred, (ii) the number of H^+^ and *e*^–^ transferred per molecule or per surface site, and (iii) changes in the composition of the molecule or surface that accompany PCET. For instance, a hydroxide-covered metal surface is reduced by 1H^+^/1e^–^ PCET with loss of H_2_O to give the bare surface.

For small-molecule PCET reactions, the H^+^/*e*^–^ stoichiometry  is always a ratio of integers by Dalton’s law (eq 5). Single-step PCET reactions are typically 1*e*^–^/1H^+^ (often H^•^ transfer^29^), and some are 2*e*^–^/1H^+^ or hydride transfers. From a molecular perspective, the Heyrovsky reaction in Scheme 1 can be viewed as hydride transfer from the surface to H^+^ in solution, forming H_2_.
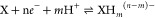
5

A valuable way to measure the H^+^/*e*^–^ ratio of a molecular half reaction (eq 5) is to determine the electrochemical potential or an equilibrium constant as a function of pH (or proton activity *a*_H^+^_ in nonaqueous media).^26^ The Nernst equation (eq 6) gives the dependence of *E* on [H^+^] (eq 6). Converting from ln to log so that log[H^+^]^−1^ = pH and entering the constants with *T* = 298 K gives eq 7, with the Nernst constant of 59.2 mV/decade. Plots of *E* versus pH thus have slopes of , where  is the proton/electron stoichiometry (eq 8). In buffered aprotic solvents, the buffer p*K*_a_ and ratio of components are involved instead of the pH.^26^ In all cases, a slope of −59.2 mV per decade in proton activity indicates equal numbers of H^+^ and e^–^ transferring  in the reaction that the *E* refers to.
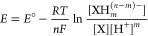
6
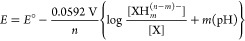
7
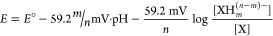
8

The redox energetics of many materials vary with pH by roughly the “Nernstian” −59 mV/pH. This is evident from Pourbaix’s *Atlas of Electrochemical Equilibria in Aqueous Solutions*, which summarizes the redox chemistry of bulk oxide and hydroxide solids in contact with aqueous solutions as a function of pH across the whole Periodic Table.^32^ Reactions at metal surfaces, such as the Volmer reaction (Scheme 1), show such a Nernstian shift with pH (see section VII below). The CB and VB energies of semiconductor interfaces also shift approximately −59 mV/pH in most cases, and this shift is usually assumed in band-energy diagrams such as Figure 3. This thermochemical property is thus common across a wide range of situations: for molecules, metals, oxides, and other materials, in solution, in bulk, and at interfaces. This commonality suggests a single origin: PCET. In fact, the definition of PCET above states that the dependence of a potential or free energy on the proton activity is *prima facie* evidence for a PCET process (though this is not the common view for semiconductor interfaces, see below).

The widespread observation of approximately −59 mV/pH shifts in potential has prompted the frequent use of the reversible hydrogen electrode (RHE) as the electrochemical reference scale (eq 9). RHE itself moves −59.2 mV/pH versus a pH-independent scale like SHE or SCE (from the Nernst equation at 298 K). Thus, many PCET potentials for molecular and interfacial reactions, including eqs 1–4, are constant versus RHE at different pHs. We have advocated for extending the use of RHE as the reference electrode for PCET in nonaqueous electrochemical media, as this connects reduction potentials to the free energy of H_2(g)_^26^ (see below). RHE can be determined experimentally in organic solvents in a wide range of media and buffers.^26,33,34^
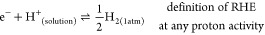
9

While the text above segued simply from molecules to materials for the ideal −59.2 mV/pH cases, there are additional complications for interfaces, bulk materials, and other extended structures. For instance, some interfaces have pH dependences that deviate substantially from −59.2 mV/pH and integer ratios (section XI, subsection iii). Interfaces often have a variety of surface sites with different properties, unlike molecules which are all identical (except for impurities). Surface reconstruction can occur *in operando* and can be driven by the binding of ligands to the surface.^35−39^ Even on highly crystalline surfaces such as Pt(111), the first H added is often not chemically the same as the last one (section XI, subsection iv).^40^

The complexities of interfaces emphasize the importance of stoichiometry beyond the proton/electron ratio. For this analysis, we will use the common H^+^/*e*^–^ PCET ratio of one, so that the species on the surface can be viewed as hydrogen atoms (H^+^ + e^–^ = H^•^). Unlike a molecule X that can add an integer number of H atoms (XH, XH_2_, etc.), on a surface any value of the surface coverage θ_H_ is possible. The maximum (saturation) coverage can be one H for every surface atom, or 1 H per 100 surface atoms, and may depend on the experimental conditions.

Direct determination of stoichiometry can be challenging because of the small number of atoms on a surface and because hydrogen is not easily detected by most spectroscopic and surface techniques. Still, there are indirect ways to quantify surface hydrogen. Titration of surface H with a soluble reagent can give accurate quantification if the chemistry is well-defined and the small amount of soluble product can be measured (cf., the reverse of eq B2 in Scheme 2). Another chemical method is temperature-programmed desorption (TPD), when H_2_ and other desorbed products are identified and quantified while heating a material.

The full surface stoichiometry is particularly challenging to establish for binary or ternary materials, MX_n_, which are of increasing interest as substitutes for noble metal catalysts.^41^ When more than one kind of element is present, the stoichiometry and structure of a catalytic surface is often not the same as the bulk. For IrO_2_ catalysis of the oxygen evolution reaction (OER), for example, well-ordered, single crystal surfaces are much poorer catalysts than amorphous, hydrated, mixed-valent surfaces.^42,43^ As noted above, the conditions being considered in this Perspective are typically corrosive, with acidic or basic solutions and with the materials undergoing chemical change.

While stoichiometry is needed to understand PCET at interfaces, stoichiometry and thermochemistry are blind to structure. A Nernstian dependence (eq 7) will usually not distinguish between H intercalated inside a material, H covalently bonded to a surface atom, or perhaps H^+^ close enough to the surface to be within the electrochemical double layer (or even a state of equilibrium among these three limiting cases).^44−47^

## V Thermodynamic Coupling between Proton and Electron Transfers

Thermochemistry is a second cornerstone of PCET chemistry. One would not analyze an ET or PT reaction without knowledge of the relevant *E*°s and p*K*_a_’s, and PCET is the same.

**Scheme 3:**
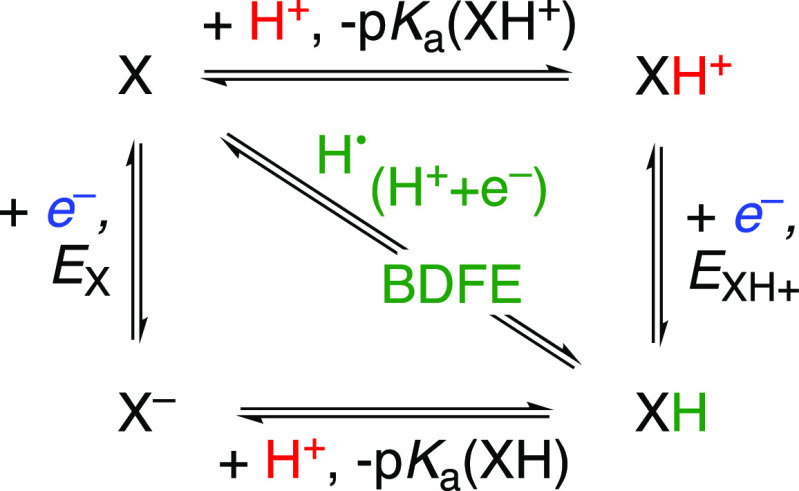
PCET Square Scheme for a Single Reagent XH, Showing ET (vertical), PT (horizontal), and Concerted PCET (Diagonal)

PCET thermochemistry is typically described using “square schemes” such as Scheme 3. The horizontal reactions are PT, and the vertical ones are ET (following the format of Pourbaix and geochemical p*E*/pH diagrams.)^32,48−50^ The diagonal represents the transfer of both *e*^–^ and H^+^, the net transfer of a hydrogen atom, and its energy is the bond dissociation free energy of X–H, BDFE(X–H) (eq 10a).
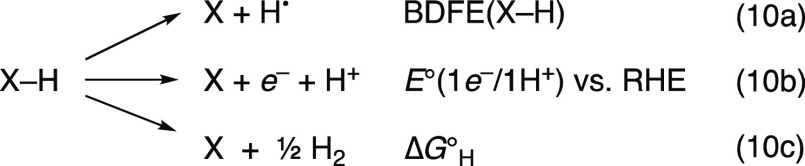


The square scheme shows five thermodynamic parameters, but only three values are independent. Δ*G*° is a state function, so the sum of energies around any closed loop back to the same point has Δ*G*° = 0 (Hess’s Law). For example, the loop around the outside of the square shows that the difference in the p*K*_a_ values must be equal to the difference in *E* values, after the appropriate conversions to Δ*G*° (eq 11; energies in kcal mol^–1^). A detailed discussion of the thermochemistry of PCET reagents is given in a recent review.^26^
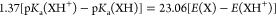
11

The BDFE (the diagonal of Scheme 3) is conceptually the sum of a p*K*_a_ and a 1e^–^ reduction potential *E*. The reactions for the p*K*_a_ and *E°* just have to sum to the diagonal (i.e., not the p*K*_a_ and *E°* of X from the top left corner). Irrespective of their origin, the *e*^–^ and H^+^ together form H^•^.^26^ Thermodynamic analyses of H-atom transfers and “separated” PCET reactions (section III) are the same. The thermochemistry being the same regardless of the *e*^–^ and H^+^ sites is key to applying BDFEs to a material interface.

The BDFE is quantitatively obtained by converting the p*K*_a_ and *E°* to free energies and adding the Δ*G*° for *e*^–^ + H^+^ → H^•^, known as *C*_G_ (eq 12; all species other than *e*^–^ are in solution).^26^ With RHE as the electrochemical reference, *C*_G_ becomes almost solvent independent, 52 ± 1 kcal mol^–1^.^26^ When the measured reduction potential is for a 1*e*^–^/1H^+^ process (eq 10b), *E*°(1*e*^–^/1H^+^ vs RHE) directly gives the BDFE via eq 13. This RHE potential (actually *eE°*) is the same as the free energy of hydrogenation per H, Δ*G*_H_ (eqs 10c and 14, because the definition of RHE is 1*e*^–^ + 1H^+^ = 1/2H_2_(g) (eq 9). Thus, the PCET approach directly connects Δ*G*_H_, a commonly used parameter in gas/solid heterogeneous catalysis, with the solution electrochemical potential *E*°(1e^–^/1H^+^ vs RHE). The three parameters in eq 10a–c are conceptually the same, differing only by constants.^26^ They are the best energetic parameters for *ne*^–^/*n*H^+^ PCET, both for molecules and for materials. We prefer BDFEs only because they more intuitively connect to molecular chemistry.
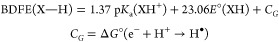
12
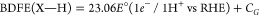
13
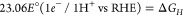
14

Most studies of semiconducting materials tacitly assume that ET and PT at the surface are independent and not coupled. The surface undergoes rapid protonation/deprotonation upon contact with the solution, which creates a positive or negative surface charge.^51^ This is independent of any ET processes. Then ET at the surface occurs without any change in the surface proton coverage. This is the traditional explanation of the Nernstian pH dependence^51^ and is discussed in section IX below. Taking PT and ET as uncoupled reactions is equivalent to assuming that the surface p*K*_a_ does not change with electron occupancy (that both sides of eq 11 are zero). This independence of PT and ET is implicit in what we refer to as the Gerischer model within this Perspective (Figures 2 and 3).

At metal surfaces, however, the Volmer, Heyrovsky, and Tafel reactions require that the ET and PT steps are closely coupled, that each electron is transferred with a proton (Scheme 1). This tight coupling means that the effective p*K*_a_ of the surface changes upon electron transfer or, equivalently, that the effective *E*° changes upon proton transfer (eq 11). Strong coupling is common in molecular PCET. For *p*-xylene, for instance, the p*K*_a_ drops by 58 units upon ET to form the radical cation, and the *E*° drops by ∼3.5 V upon deprotonation to the carbanion.^26^ This strong ET/PT coupling makes PCET different than separate ET + PT. Strong coupling PCET is inherently different from the electron-focused “Gerischer model”.

## VI Analogy between PCET and Lithium-Ion Batteries: The “Battery Model”

**Scheme 4:**
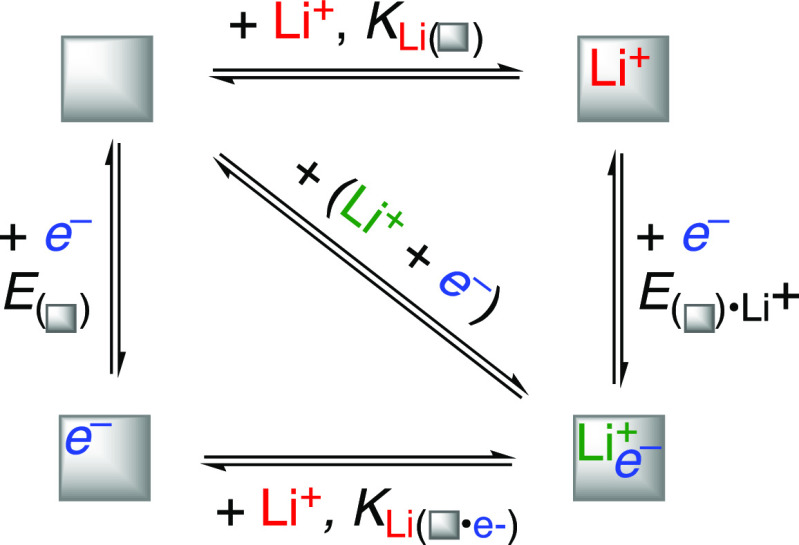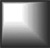 Battery Anode or Cathode Adapted from ref (53). Copyright 2019 American Chemical Society.

Lithium-ion batteries (LIBs) are analogous to PCET in that both involve cation transfer coupled to ET. Writing a “square scheme” for LIBs, deconstructing the ET and Li^+^-binding steps into individual *e*^–^ and Li^+^ transfers, makes this analogy explicit (Scheme 4). Scheme 4 is directly parallel to Scheme 3 for PCET. In LIBs, every *e*^–^ transferred is accompanied by Li^+^ transfer, in both charging and discharging, at both the anode and the cathode. It is not typically valuable to consider the transfer of just an *e*^–^ or just a Li^+^ in LIBs because their transfers are tightly coupled. These issues are common to most batteries, so we call this the “battery model.”^52,53^

The strong *e*^–^/Li^+^ coupling in the battery model requires that the battery electrode material has a larger affinity to bind or intercalate a Li^+^ after an *e*^–^ is added.^53^ Using the quasi-equilibrium constants in Scheme 4, *K*_Li(■•*e*−)_ must be larger than *K*_Li(__■)_ (the solid square indicates the material). In a computational example, the *K*_Li_ for Li^+^ intercalation into small TiO_2_ clusters was calculated to increase by 10^8^ upon addition of 1*e*^–^.^54^ The shift in *K*_Li(__■__)_ is the same as the change in *E*_(__■__)_ upon Li^+^ binding because the analysis is exactly parallel that for PCET (eq 11). The Li electrode reference used for batteries (eq 15) is conceptually the same as RHE. The battery model requires that the properties of the material change upon addition of *e*^–^ or X^+^, in contrast to the independence of ET and PT in the Gerischer model.
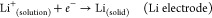
15

The battery model depends only on the 1:1 stoichiometry of the reaction. It is independent of the details of the material. It is the same for Li^+^ intercalated into a solid, as for graphite anodes and for oxide cathodes such as Li_*x*_CoO_2_, or for Li^+^ added to the surface of a Li metal anode. The 1:1 stoichiometry is often favored because it achieves local charge balance, in LIBs as in the Volmer, Heyrovsky, and Tafel reactions.

An important implication of this analysis is that a material that undergoes PCET or Li^+^-coupled ET cannot be characterized by a single work function, Fermi energy, or valence-conduction band energies. These terms reflect primarily the energy of an electron. The electronic energies must change upon addition of the cation, and the cation affinity must change upon addition of *e*^–^, or else the reaction would not be a coupled transfer. Thus, the ubiquitous use of such single, electron-only parameters to characterize interfacial redox reactions is inappropriate when the stoichiometry is 1e^–^/1H^+^ (or 1*e*^–^/1M^+^). With this stoichiometry the measured energy is not an electronic energy, it is the surface–H BDFE, and the Δ*G* to add *e*^–^ + H^+^ or an H atom (or a Li atom).

## VII PCET at Metal Interfaces

Protonation of metal surfaces is established as a 1*e*^–^/1H^+^ PCET process, the Volmer reaction (Scheme 1).^55−57^ From one perspective, the reaction is PCET because bringing a 1+ charge to the surface of a metal will generate a 1– image charge in the metal, which requires one electron.

**Figure 4:**
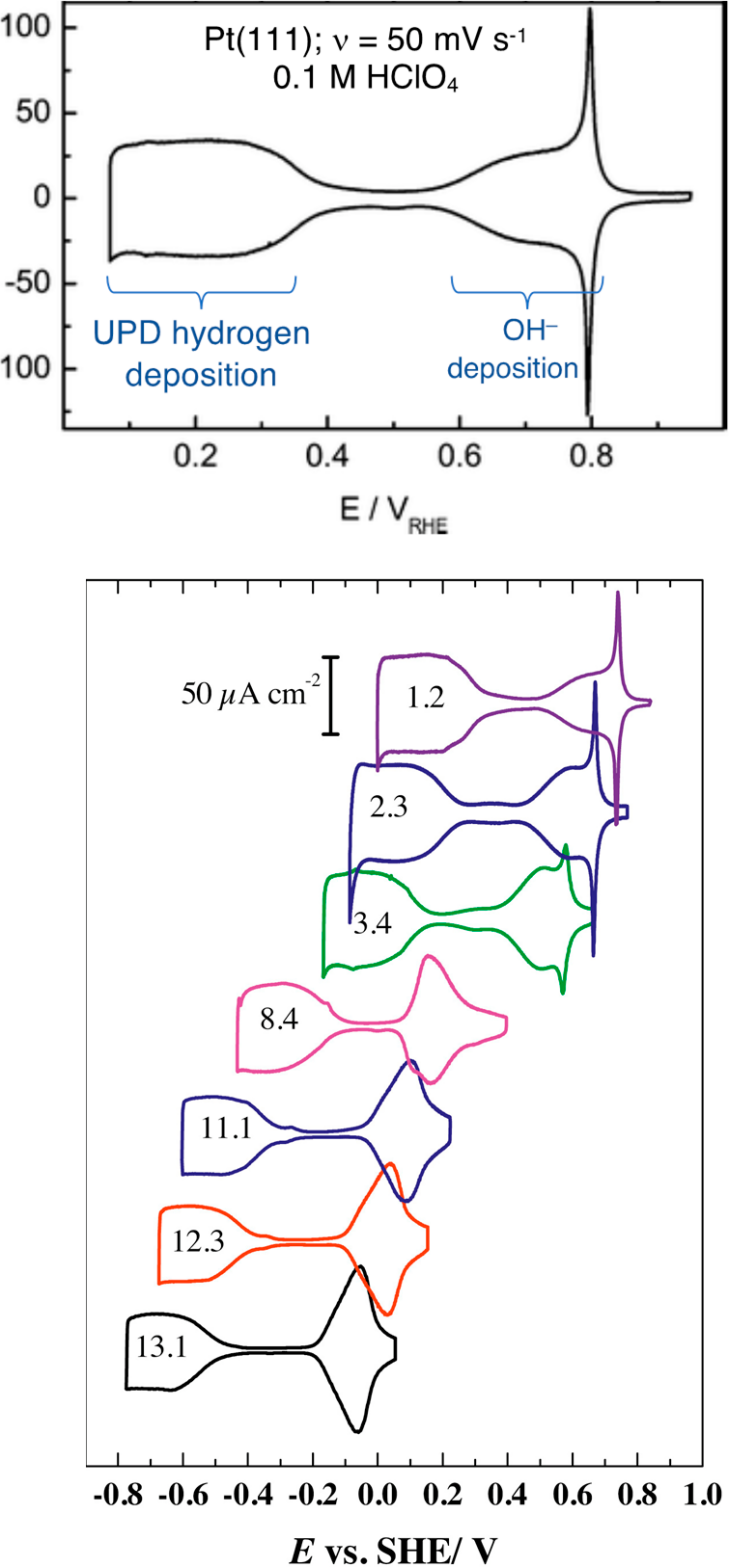
Top: Cyclic voltammogram of Pt(111) in 0.1 M HClO_4_ sweep rate 50 mV s^–1^, with the UPD-H region labeled. Adapted with permission from ref (64). Copyright 2008 Royal Society of Chemistry. Bottom: Cyclic voltammetry of a Pt(111) electrode at different solution pH’s (scan rate: 50 mV s^–1^). The wave for UPD hydrogen is the shape at the left in each CV, with the pH inscribed inside. Reprinted with permission from ref (58). Copyright 2015 Elsevier.

One of the best studied cases of metal hydrogenation is the formation of underpotential deposited hydrogen (UPD-H) on platinum.^56^ CVs of Pt(111) single crystal faces show reversible Faradaic current between +0.3 and 0 V versus SHE at pH 1 (Figure 4, top).^58^ This wave corresponds to the formation of UPD-H by the Volmer reaction. The broad CV peak shifts approximately −59 mV/pH over the normal pH range (Figure 4, bottom), so its potential is essentially constant versus RHE. On the basis of the Nernstian shift and other studies, the reaction occurring has the 1:1 *e*^–^: H^+^ Volmer stoichiometry.^56^ In addition, UPD-H is the same as the H added to Pt with H_2_, which must be 1*e*^–^/1H^+^. This well studied system is thought to be representative of all electrochemical additions of protons to metal surfaces (“proton discharge”). As discussed in section XI, subsection iv below, there is also a second kind of H on Pt(111): overpotential hydrogen (OPD-H), the catalytically active form.

The conclusion of PCET at metal surfaces has interesting implications. Metal surfaces follow the battery model, not the Gerischer model. In the Volmer reaction, the approach of an H^+^ to the surface draws an e^–^ from the potentiostat, whether a surface monolayer or intercalated H are formed (UPD-H on Pt or H addition to Pd). Further implications are developed in the next section.

## VIII Equivalence of Electrochemical (Faradaic) and Non-Faradaic PCET

Interfacial *ne*^–^/*n*H^+^ PCET reactions are the same whether they occur by a chemical reaction or an electrochemical process. The same Pt–H is made by the electrochemical Volmer half reaction and by the chemical reaction of the Pt surface with H_2_. The reduction of ethylene to ethane can be accomplished electrochemically with 2*e*^–^ + 2H^+^ or chemically with H_2_. The same surface intermediates are involved (under similar conditions).

The equivalence of the chemical and electrochemical processes extends to their thermodynamic parameters. For an *ne*^–^/*n*H^+^ PCET reaction referenced to RHE (eq 16), the electrochemical potential *E*_RHE_ (actually *eE*_RHE_) is the same as the chemical free energy of hydrogenation (per H; eq 17). This identity holds in any solvent, at any solution proton activity, and at any X/X_*n*_ concentration ratio, because RHE connects *e*^–^ + H^+^ with H_2_(g), which is the common “anchor”: eq 16 – eq 9 = eq 17. A longstanding example is the connection between the Volmer reaction and surface–H binding free energies (via eqs 12–14 above).^56,59−63^
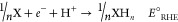
16
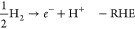
–9
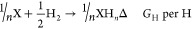
17

The specific equality of *eE*_RHE_ and Δ*G*_H_ does not hold when there are unequal numbers of protons and electrons, for instance for hydride transfer reactions (2*e*^–^/H^+^ = H^–^), or when any reference electrode other than RHE is used.

The unusual feature of a *ne*^–^/*n*H^+^ PCET half reaction is that it is charged-balanced (eq 16). Fundamentally, electrochemistry is about the movement of charges, and *ne*^–^/*n*H^+^ transfers from a solution to an interface do not involve the net movement of charge. This makes the correspondence with a chemical (whole) reaction more direct.

A central parameter in outer-sphere ET reactions at electrodes is the electrochemical potential of the electron . In general, the electrochemical potential for a species *i* with charge *z*_*i*_ (*μ̅*_*i*_, eq 18) is the chemical potential *μ*_*i*_ (eq 19) plus the work to bring the species from standard state to an electrical potential ϕ.^8^ For example, the Volmer reaction at equilibrium at a Pt surface can be written in terms of the electron and proton electrochemical potentials  and  (eq 20).  and  are intuitive experimental variables: the potentiostat adjusts , and added reagents adjust . This separability is in the definition of the chemical potential because the partial derivative is explicitly with the amounts of the other species held constant (*∂n*_*j*≠*i*_ = 0, eq 19). However, the stoichiometry of the Volmer reaction (eq 21) requires that  and  be coupled.
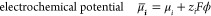
18
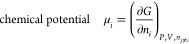
19
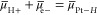
20
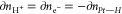
21

The e^–^/H^+^ coupling in a PCET reaction means that both  and  are needed to describe the thermochemistry. The equivalence of *E*°(1*e*^–^/1H^+^ vs RHE) and Δ*G*_H_ shows that PCET cannot be developed using primarily the electrochemical potential of the electron, . In general, measured electrochemical cell potentials report on the overall reaction that is occurring (*E* = Δ*G*/*nF*), not simply on . For example, the potential for oxidizing copper metal to [Cu(NH_3_)_4_]^2+^ includes the removal of Cu from the metal, its oxidation to Cu^2+^, and binding of NH_3_ ligands. Similarly, PCET includes ET and PT, so  is always paired with .

## IX PCET at Semiconductors

The potentials versus NHE for most semiconductor/aqueous interfaces shift −59 mV/pH (see also section XI, subsection iii below). This shift means that the pH or proton activity should be specified in any potential measurement. A Nernstian shift is often implicit in diagrams such as Figure 3, for instance being used to extrapolate measured values to pHs where the materials are unstable or soluble (e.g., ZnO and Fe_2_O_3_ at low pH). The assumption of a Nernstian shift should perhaps be explicit when the semiconductor potentials are lined up with the H_2_/H_2_O and O_2_/H_2_O couples (eqs 1 and 2), and when potentials are given versus RHE.

The Nernstian −59 mV/pH shift of the band edge or flat-band potentials is traditionally explained by changes in surface charge.^51^ At lower pH, surfaces are more protonated and therefore more positively charged, so it is easier to add electrons (more positive potentials). The Nernstian slope is predicted when the proton surface coverage is proportional to the solution proton activity (pH or *a*_H^+^_).^51^ As noted above, this model assumes independent ET and PT, as the PT happens before any current is passed.

**Figure 5:**
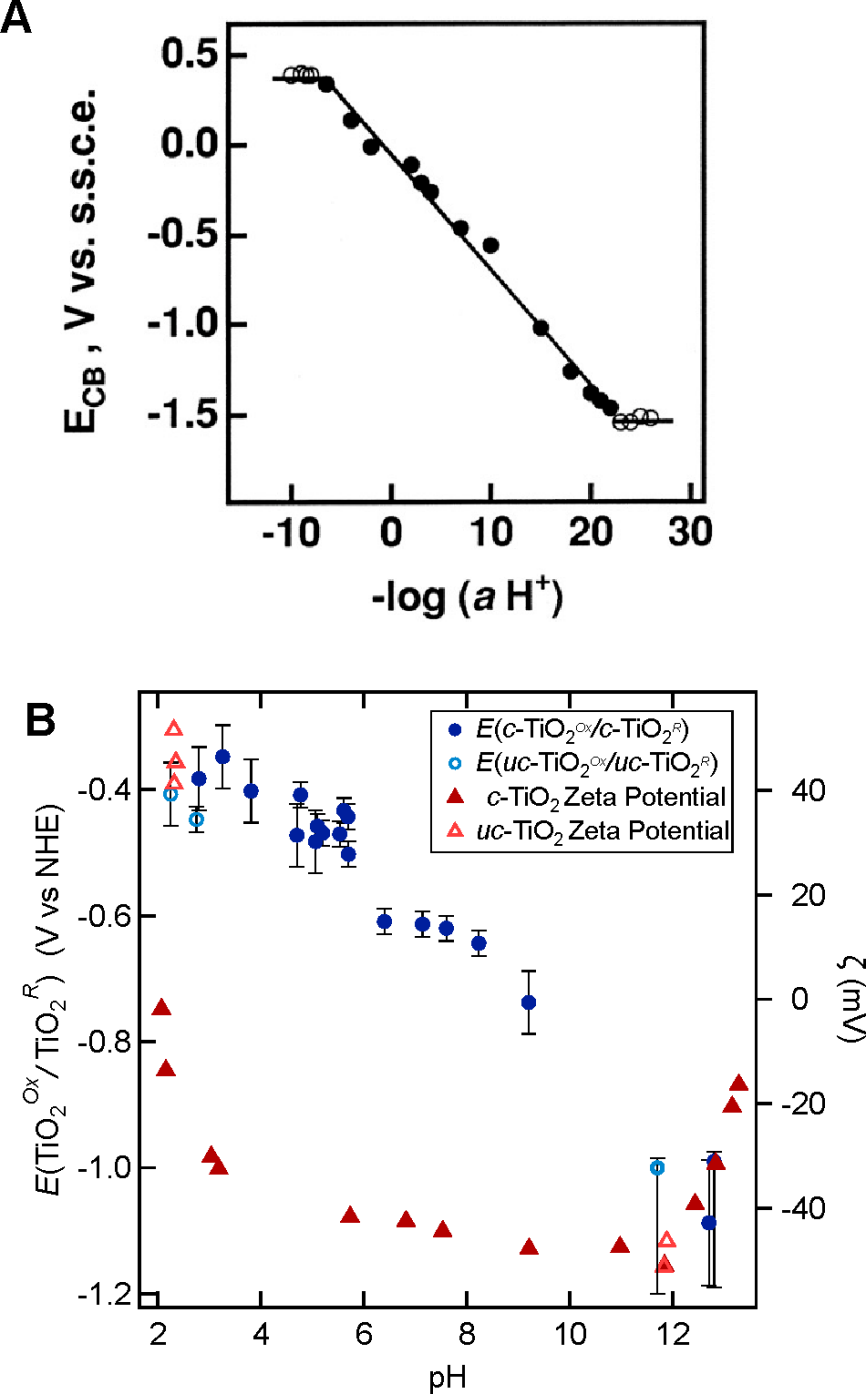
(A) Dependence of reflectance-derived conduction band edge energy on log(proton activity). Reproduced from ref (65). Copyright 1999 American Chemical Society. (B) Plot of *E*(TiO_2_/TiO_2_·*e*^–^,H^+^) (left axis, circles) and zeta (ζ) potential (right axis, triangles) vs pH, for colloidal TiO_2_ nanoparticles. Data are mostly for citrate-capped TiO_2_ [*c*-TiO_2_, blue ● (*E*) and red ▲ (ζ)] with a few values for “uncapped”-TiO_2_ [*uc*-TiO_2_, blue ○ (*E*) and red △]. Reproduced from ref (71). Copyright 2022 American Chemical Society.

An early study that questioned the surface charging explanation examined the variation in the TiO_2_ conduction band energy over 10^40^ in *a*_H^+^_ (Figure 5A).^65^ For the middle 25 orders of magnitude, the *E*_CB_ varied 64 mV per −log(*a*_H^+^_). The surface H^+^ concentration cannot change by 10^25^, as there are not enough surface sites (∼10^14^ cm^2^).^66^ With the use of an electrochemical quartz crystal microbalance, reduction of the TiO_2_ caused twice as much mass gain in D_2_O as in H_2_O. The authors therefore suggested that “electrochemical generation of Ti(III) trap sites ... is accompanied quantitatively by proton intercalation”, in other words, PCET.^65^

A growing body of research on semiconductor/solution interfaces indicates that the ca. −59 mV/pH shift is due to strongly coupled 1:1 ET and PT. Reductive proton intercalation into hydrous phases of WO_3_, RuO_2_, and other oxides on electrodes is well established.^67^ Colloidal TiO_2_ nanoparticles (NPs) have a roughly Nernstian shift in their apparent reduction potential,^68−71^ but their ζ potentials, which should correlate with particle surface charge,^72,73^ have a very different pH dependence than *E*_TiO_2__ (Figure 5B).^71^ Oxidation of reduced TiO_2_ NPs with KI_3_ gave a drop in pH, implicating a loss of H^+^ to the solution, with a stoichiometry of slightly more than one H^+^ per *e*^–^.^71^ Thus, the thermodynamic preference of TiO_2_ is to stoichiometrically couple *e*^–^ and H^+^.

ZnO NPs suspended in THF/toluene similarly added 0.9 e^–^ from a soluble reductant for each H^+^ added to the organic solution.^74,75^ Adding Na^+^ ions had a similar effect, while Mg^2+^ and Ca^2+^ gave ∼1.4 e^–^ per M^2+^.^76^ This similarity of PT and metal cation transfer and the larger stoichiometry for M^2+^ demonstrates the importance of charge balance in these reactions.

Flat-band and band-edge energies for semiconductor/solution interfaces are commonly measured using Mott–Schottky plots. This approach requires that at high measurement frequencies, only electrons move. Analyzing the same experimental data with a PCET (battery) model would be of interest. Mott–Schottky analyses can also be problematic when surfaces are not planar and uniform, when there are surface states, and when multiple processes occur at the electrode.^77,78^ These and other effects contribute to the large variation in energies reported for nominally the same material, ∼0.5 V for TiO_2_.^79^

Nickel oxide electrodes in aqueous solutions have long been known to have two Faradaic oxidations that shift approximately −59 mV/pH and that are best described as PCET (eqs 22 and 23 and the TOC image).^80,81^ We have recently found that a NiO electrode also shows Faradaic waves in buffered acetonitrile or dimethylformamide (DMF) solutions.^82^ The waves shift ca. −59 mV per unit change in buffer p*K*_a_ for 1:1 buffers. The NiO–H BDFEs from eq 12 were confirmed by equilibration of the electrodes with a soluble phenoxyl radical.^80−82^ Remarkably, the BDFEs for the NiO electrodes are the same in different solvents and with a variety of buffers.^82^ In contrast, electron-only or proton-only energies are very solvent-dependent because they involve movement of charge. Molecular BDFEs are also known to be solvent-independent.^26^ The constancy with reaction conditions is one of the reasons why BDFEs should be the preferred energy metric for solid/solution interfaces as well as molecules.^82^
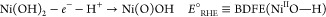
22
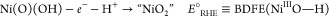
23

The various examples in this section support the general conclusion that a Nernstian potential with pH or *a*_H^+^_ is due to a stoichiometric and thermodynamic coupling of ET and PT, 1e^–^/1H^+^ PCET.

For nonoxide semiconductors, the literature on the aqueous energetics is more limited. Studies of silicon, metal-chalcogenide, and metal-pnictide materials typically show approximately −59 mV/pH shifts.^83−86^ These surfaces are easily oxidized, and the Nernstian shifts may be a result of the surface oxide formed. More work in this area would be valuable, as some of these materials are being explored as earth-abundant replacements for precious metal catalysts.^41^

## X BDFEs Provide a Unified View of Interfacial PCET

**Figure 6:**
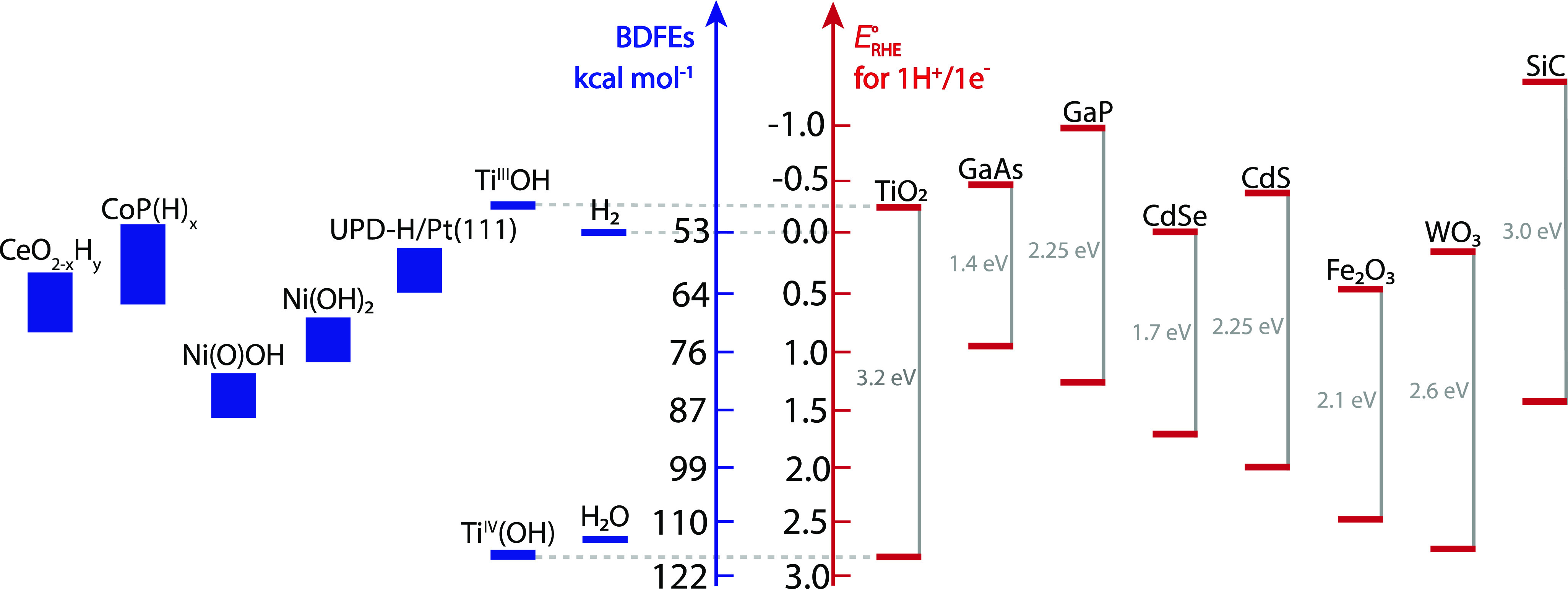
Recasting a traditional plot of conduction and valence band energies such as Figure 3 to reflect the likely 1*e*^–^/1H^+^ nature of the reduction potentials and to show surface–H bond dissociation free energies (BDFEs) for different materials. The heights of the blue boxes on the left side of the diagram show the measured BDFE range for each material.

The conclusion that PCET is the norm for redox reactions at a solid/protic solution interfaces prompts a re-examination of the focus on semiconductor band edge energies (e.g., Figure 3). When the *E* are for 1*e*^–^/1H^+^ processes, the energies are better recast as surface–H BDFEs (eqs 10 and 13 above), as shown in Figure 6 on the left in blue. The right side has the more traditional band potentials in burgundy, now labeled *E°*_RHE_ for 1*e*^–^/1H^+^. H_2_ and RHE are the common anchor on the two scales. The BDFE and *E°*_RHE_(1*e*^–^/1H^+^) are the same (section V above), differing only by the constant *C*_G_ = 53 kcal mol^–1^ in water (Δ*G*° for 1/2H_2(g)_ → H^•^_(aq)_, eq 13).^26^

**Figure 7:**
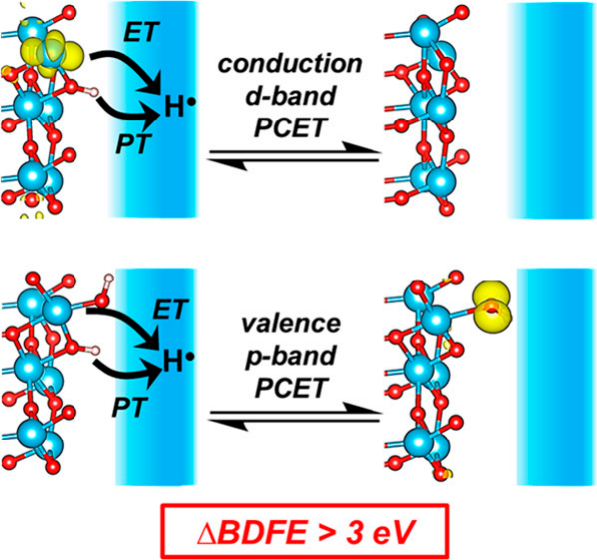
PCET from (top) a Ti d-band state, formally [TiO_2_]^−^[H]^+^ → [TiO_2_]^0^ + (H^+^ + *e*^–^), and (bottom) an O p-band state, [TiO_2_]^0^[OH]^−^[H]^+^ → [TiO_2_]^0^[O^•^] + (H^+^ + *e*^–^). The converged geometries for each structure have a yellow isosurface representing the charge density associated with the Ti^3+^ polaron (top) or valence band hole (bottom).^87^ Reproduced from ref (87). Copyright 2021 American Chemical Society.

The two entries for TiO_2_ on each side of Figure 6, connected by dashed lines, represent different views of the PCET model. The upper burgundy bar originally indicated the potential to add an electron to the bottom of the CB. In the PCET version, this is the potential to add *e*^–^ + H^+^. The product of *e*^–^ + H^+^ (or H^•^) addition to TiO_2_ can be viewed as a Ti^III^(OH) site, in a localized limit. However, the *e*^–^ could also be in the CB or (most likely) delocalized over a few Ti ions; as emphasized above, the thermochemistry is consistent with different structures. The O–H BDFE for this state is low because the OH is fairly acidic and the electron is easily removed (it lies at fairly high energy). A recent study computed this BDFE to be ∼39 kcal mol^–1^ at a TiO_2_ slab (varying slightly between different sites).^87^ This value is close to an experimentally measured value of 49 kcal mol^–1^ for colloidal TiO_2_ NPs [Figure 7, top blue bar for Ti^III^(OH)].^71^

The lower burgundy bar for TiO_2_ represents the energy of a hole (*h*^+^) at the top of the oxide VB, formed by removal of H^•^ from a Ti^IV^(OH) species. Cleaving this O–H bond is very difficult, with a high BDFE of ∼120 kcal mol^–1^, because the *h*^+^ formed is at high energy (the *e*^–^ in the VB is tightly bound).^87^ The Ti^IV^O–H value is close to that for the first O–H BDFE in water,^26^ which is why the reactivity of the TiO_2_ hole states (“Ti^IV^O^•^”) is similar to that of hydroxyl radical (HO^•^): they react to form O–H bonds of similar strength. The Ti^III^O–H BDFE is less than half that in water but close to values for molecular Ti^3+^(OH_2_) complexes.^88^

The difference between the BDFEs of these otherwise similar O–H bonds on a TiO_2_ surface is a remarkable ∼80 kcal mol^–1^ (∼3.5 eV). While it is tempting to connect this difference with the TiO_2_ band gap of similar magnitude, the computations show that a number of terms contribute.^87^ This study shows the complexity of computing surface–H bond strengths when the nature and density of defect states are not known.

The generality of the BDFE approach is illustrated by the inclusion of materials on the left side of Figure 6 that are not semiconductors. BDFE values are included for H_2_ (per H) and H_2_O, and many other molecules could be added.^26^ UPD-H on Pt(111) is included, and again values for other metals could be added. The height of the blue bar for UPD-H/Pt and for other materials indicates the range of surface–H BDFEs, as discussed in section XI, subsection iv below. Nickel oxide, CoP, and CeO_2–*x*_ have electronic structures more complex than a simple semiconductor but are easily included in the BDFE chart. This representation is appropriate for any material or molecule that has an X–H bond.

## XI Generality, Limitations, Exceptions, and Complexities of the 1*e*^–^/1H^+^ PCET Model

### i Generality and Scope

Solid materials accepting or donating electrons generally charge-balance much of the electron flow. When protons are present, this normally leads to *ne*^–^/*n*H^+^ PCET. The same PCET framework applies to semiconductor and metal interfaces. PCET is one example of ion-coupled electron transfer at interfaces.

On the basis of results with NiO electrodes,^89^ surface–H BDFEs seem to be independent of medium (solvent and buffer). This is also a property of molecular BDFEs.^26^ In contrast, electron-focused parameters such as band-edge energies vary substantially with the medium. Therefore, BDFEs are the better parameter to compare systems in different media and to guide the development of devices in novel media, including those where pH (*a*_H^+^_) is not well-defined.

Still, the discussion here has been limited to the stoichiometry and thermochemistry of PCET at interfaces. Connections of these with the kinetics and mechanism of surface hydrogen reactivity are just beginning to be understood. Molecular 1*e*^–^/1H^+^ PCET reactions can occur by the concerted transfer of the two particles in a single elementary kinetic step or by stepwise ET-then-PT or PT-then-ET.^90^ The same will likely be found for interfacial reactions.

A thermochemical view is likely most appropriate in the long time scale and high concentration limits. For example, photoinduced charge injection into nanoscale TiO_2_ in a dye-sensitized solar cell occurs on time scales from femtoseconds to microseconds. Hours of continuous irradiation forms a material with ca. 10^18^*e*^–^ cm^–3^ (*∼*1e^–^ for every 3000 Ti ions),^91^ and both H^+^ and Li^+^ likely intercalate to charge balance the electrons.^92^ On ultrafast time scales, ET could occur prior to cation transfer, though ultrafast concerted PCET is known for molecules^93,94^ and interfaces.^95,96^ Perhaps ET is decoupled from PT at low doping levels and at short times, in which case the Gerischer model is most appropriate. Still, discussions of pure ET in this case should not use long-time scale thermodynamic values determined in the PCET regime.

### ii Limitation of the Battery Model: Chemical (Faradaic) vs Physical (capacitive) Processes

This Perspective is about chemical and electrochemical reactions of solids that are in contact with a protic solution. A solid can also undergo physical processes under the discussed conditions, such as developing a surface charge. This perhaps simplistic separation between chemical and physical processes roughly parallels the electrochemical distinction between Faradaic and capacitive processes. The surface charge of the material creates an electrochemical double layer (EDL) and has a capacitance. In general, applying a potential to a redox-active electrode will lead to both PCET (or other ion-coupled ET) and EDL charging.

In many electrochemical experiments, the Faradaic (PCET) current is larger than the capacitive (EDL) current. This is illustrated in the CV of Pt(111) (Figure 4, top) where the capacitive current between 0.4 and 0.6 V is smaller than the Faradaic currents on either side. Still, to obtain the wide range of potentials in Figure 4, bottom, requires polarizing the electrode by electron transfer (balanced only the EDL), as shown by measurements of the potential of zero free charge (PZFC).^58,97,98^ Semiconductor electrodes can have similar capacitive and Faradaic currents, particularly for high-surface area materials with low fractions of electroactive material, as in the detailed study of mesoporous TiO_2_ by Balland, Limoges, and Costentin.^99,100^ Capacitive, not ion coupled, current is clearly important in electrochemical processes, in addition to PCET.

Still, from a chemical standpoint, Faradaic and non-Faradaic PCET reactions are of greater interest because they are the ones that drive catalysis, that lead to corrosion, etc. The materials in Figures 3 and 6 are different because of their distinct Faradaic behaviors.

### iii Exceptions: Non 1*e*^–^/1H^+^ Behavior

Quite a few oxide interfaces have energetics that shift more than the Nernstian −59 mV/pH. These “super-Nernstian” shifts, often −90 or even −120 mV/pH, are particularly common for hydrated oxides grown on a metal.^67,101−105^ Cyclic voltammograms of aqueous colloidal IrO_2_ NCs showed 70 or 75 mV per pH shifts for the Ir^V/IV^ and Ir^IV/III^ couples, except for the latter couple between pH 6–13 that shifted 116 mV/pH.^104^ Hydrated oxides can be the most catalytically active forms of these materials, and they can be good pseudocapacitor materials.^42,67,106,107^ Shifts of −90 or −120 mV/pH imply 1.5 or 2 protons per electron added, which by itself would not give charge balance. Perhaps the excess proton charge is balanced by coaddition of electrolyte anions. There might perhaps be an analogy with the apparent noninteger proton/electron ratios in proteins, due to redox changes causing small shifts of p*K*_a_’s and protonation states in multiple sites.^108^ Understanding the super-Nernstian hydrous oxides has been challenging because of the complexity of their structures and variability of their compositions.^101^

To our knowledge, there are only four examples of semiconductor/aqueous interfaces that do not shift with pH: the fully methyl-covered surface of Si(111),^83^ fully methyl- or allyl-covered GaP(111),^109,110^ and NiO coated with a membrane-mimicking dye.^111^ These surfaces have hydrophobic coverings with no sites for protons or H-atoms to bind. The Si and GaP examples slowly develop oxide coatings upon extended exposure to air and water, and the approximately −59 mV/pH behavior returns. Non-Nernstian behavior could be a valuable tool for optimizing solid/liquid interfaces for catalysis and other applications, as it partially decouples the surface energetics from the substrate energetics (e.g., eqs 1–4).

### iv Complexity: Nonideal Surface Isotherms

In a solution of a pure material all of the molecules have the same properties, but interfaces are more complex. Surfaces typically have chemically different surface sites, including different crystal facets, intrinsic defects (terraces, steps, adatoms, vacancies, etc.), extrinsic defects (impurity atoms), and differences in surface stoichiometry. Even on a well-ordered crystalline surface, adsorbates interact with each other, often strongly, which causes differences in their properties at low and high surface coverages. These effects create a range of surface properties, including a range of surface–H BDFEs.

The nonideality of adsorbates is well-known in the surface science and surface electrochemical literature, where they are typically described by isotherms.^112−114^ An ideal adsorbate follows the Langmuir isotherm and shows a Gaussian feature in cyclic voltammograms with a fwhm of 90 mV. The CV for UPD-H Pt(111), however, has a width of >300 mV and deviates from a Gaussian shape (Figure 4). Since all the sites are the same on Pt(111), the broadening must be due to adsorbate interactions that vary with surface coverage. From a BDFE perspective, the >300 mV CV wave for UPD-H indicates a ca. 5 kcal mol^–1^ range of Pt–H BDFEs. Platinum surfaces also bind a second class of surface H: overpotential deposited hydrogen (OPD-H).^56,62,115−118^ As the name suggests, OPD-H is over the potential to make H_2_. It forms H_2_ very quickly, making it difficult to study. OPD-H occupies different surface sites than UPD-H (atop vs 3-fold) and has weaker Pt–H bonds. There are thus two very different classes of H on Pt(111), with only OPD-H active in catalysis. And this is among the simplest redox-active interfaces, with a flat, highly ordered surface and a monatomic adsorbate!

**Figure 8:**
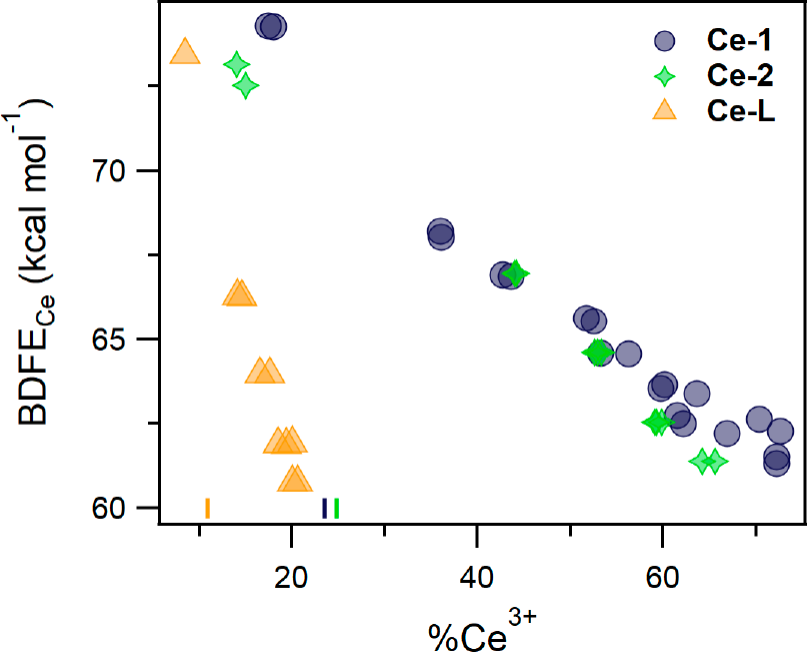
Plot of BDFE_Ce_ vs the %Ce^3+^ of oleate-capped ceria NPs suspended in THF. The BDFEs were determined by equilibration with various hydroquinones. The %Ce^3+^ values were determined by XAS and mass balance. Ce-1 (blue ●), Ce-2 (green stars), and Ce-L (orange ▲) are different batches of NPs with average diameters of 1.8, 1.9, and 4.0 nm, respectively. Reproduced from ref (119). Copyright 2021 the American Chemical Society.

Heterogeneous catalysts are often nanoscaled, high-surface-area materials that have much more complex surfaces than Pt(111). To model these, our lab has been studying colloidal nanoparticles such as oleate-capped 2 nm cerium oxide (ceria) nanoparticles suspended in THF.^119^ Surface O–H BDFEs were measured by equilibration with molecular reagents such as quinone/hydroquinone redox couples. These studies revealed a large range of Ce^III^O–H BDFEs, ≥13 kcal mol^–1^ (0.6 eV). The BDFEs became weaker as the H-atom surface coverage increased, as more of the surface became reduced to Ce^III^OH (Figure 8).

A range of thermochemistry can complicate the use of BDFEs in linear free energy relationships (LFERs), often called Bro̷nsted–Evans–Polanyi (BEP) relationships. Such correlations have been extensively applied to stoichiometric and catalytic reactions.^112−114,120−124^ While the surface-science literature describes cases where nonideal isotherms have only a small effect on the kinetics,^112^ that seems unlikely to hold in general. BEP relationships should not hold across different types of surface sites, which are common even for simple materials. For instance, IR spectra identify (Si)_3_SiH, (Si)_2_SiH_2_, and (Si)SiH_3_ sites on most H-terminated silicon surfaces.^125,126^ The striking difference in reactivity between UPD-H and OPD-H on Pt(111) surfaces is a dramatic deviation from a BEP relationship.

For most interfaces, the range of thermochemistry and the different types of surface sites have not been identified experimentally. Our study of high-surface-area cobalt phosphide (CoP) revealed a wide experimental range for surface–H BDFEs, ∼16 kcal mol^–1^ (0.7 eV).^127^ The BDFEs were estimated by titration of hydrogen-covered, high-surface area CoP with H-atom acceptors (Y in eq B2, Scheme 2). However, the experiments did not identify the surface sites, which are likely quite varied based on computational studies that implicate P–H, Co_*n*_–H, and bridging sites for H.^128^

**Figure 9:**
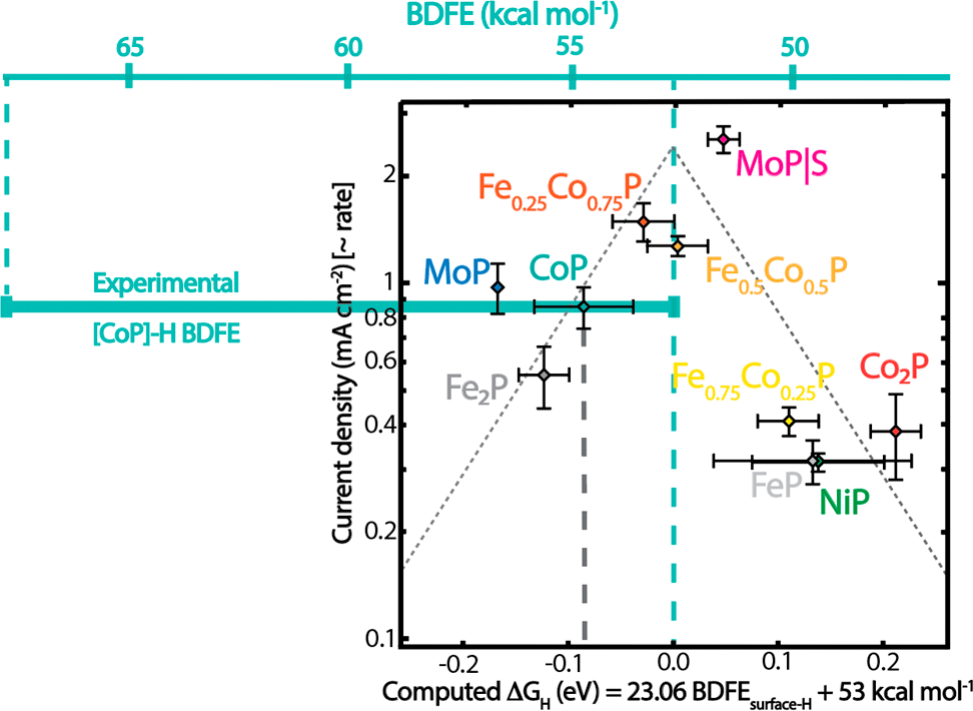
Experimental range of surface–H BDFEs on CoP (thick teal bar)^127^ superimposed on top of a volcano plot for HER by transition metal phosphides.^130^ The square volcano plot, adapted from Figure 2D in J. Kibsgaard et al.,^130^ shows experimental catalytic current density versus computed H-binding energies for crystalline surfaces. The computed BDFE/Δ*G*_H_ for CoP is indicated by the vertical black dashed line. The BDFE scale at the top parallels the bottom Δ*G*_H_ scale using BDFE = Δ*G*°_H_ + *C*_G_ (eqs 13 + 14).

Figure 9 superimposes the experimental BDFE range^127^ on top of a volcano plot^121,123,129^ for electrocatalytic hydrogen evolution (HER) by transition metal phosphides.^130^ The volcano plot shows the dependence of the catalytic HER currents at 100 mV overpotential on values of Δ*G*°_H_ computed for model single-crystal surfaces. The experimental range for H just on CoP is wider than the range of computed Δ*G*°_H_ values for all of the phosphides considered. While the volcano plot captures some general trends, going beyond this first-order treatment will require understanding the broad isotherm for hydrogen adsorption.

## XII Conclusions

This Perspective argues that PCET is a very common redox reaction at interfaces of materials and protic solvents. A survey of many different systems is presented, starting with the paradigmatic Volmer reaction, the addition of *e*^–^ and H^+^ to a metal to form a surface–H bond. The driving force for PCET is to maintain charge balance.

The Perspective develops parallels between PCET and lithium-ion batteries (LIBs), in which a Li^+^ rather than H^+^ accompanies each electron. PCET reduction potentials processes versus RHE are analogous to battery half-cell potentials versus the Li^+^/Li electrode. PCET processes with 1*e*^–^/1H^+^ stoichiometry are well described by the surface–H bond dissociation free energy (BDFE), and LIBs can be viewed as transfers of lithium atoms. The surface–H BDFE is equivalent to the free energy of hydrogenation per H atom, (Δ*G*°_H_), and to *E*°_RHE_ for a 1*e*^–^/1H^+^ half-reaction.

The PCET approach applies to metal and semiconductor interfaces, so that these and molecules can all be put on the same BDFE scale (Figure 6). This Figure is a re-evaluation of long-standing analyses of semiconductor interfaces using band-edge energies. Since PCET reactions involve both *e*^–^ and H^+^ transfers, electron-focused energy parameters such as band energies, Fermi energies, and electrochemical potentials are not sufficient. The PCET approach could provide a bridge between band structures and the use of H binding energies as catalytic descriptors. However, the complexity of H binding to real surfaces may not be fully captured in a single-descriptor model (Figure 9).

This Perspective shows that bringing a molecular PCET approach to interfacial redox reactivity provides new insights to tackling challenges in catalysis and other areas.
